# The Mincle ligand trehalose dibehenate differentially modulates M1‐like and M2‐like macrophage phenotype and function via Syk signaling

**DOI:** 10.1002/iid3.186

**Published:** 2017-07-19

**Authors:** Kristel Kodar, Jacquie L. Harper, Melanie J. McConnell, Mattie S. M. Timmer, Bridget L. Stocker

**Affiliations:** ^1^ School of Chemical and Physical Sciences Victoria University of Wellington PO Box 600 Wellington New Zealand; ^2^ Malaghan Institute of Medical Research PO Box 7060 Wellington New Zealand; ^3^ School of Biological Sciences Victoria University of Wellington PO Box 600 Wellington New Zealand

**Keywords:** Macrophages, macrophage repolarization, M1‐like, M2‐like, trehalose dibehenate

## Abstract

**Introduction:**

Macrophages play a significant role in the progression of diseases, such as cancer, making them a target for immune‐modulating agents. Trehalose dibehenate (TDB) is known to activate M1‐like macrophages via Mincle, however, the effect of TDB on M2‐like macrophages, which are found in the tumor microenvironment, has not been studied.

**Methods:**

qRT‐PCR, flow cytometry, cytokine ELISA, and Western Blotting were used to study the effect of TDB on GM‐CSF and M‐CSF/IL‐4 derived bone marrow macrophages (BMMs) from C57BL/6 and Mincle^−/−^ mice.

**Results:**

TDB treatment up‐regulated M1 markers over M2 markers by GM‐CSF BMMs, whereas M‐CSF/IL‐4 BMMs down‐regulated marker gene expression overall. TDB treatment resulted in Mincle‐independent down‐regulation of CD11b, CD115, and CD206 expression by GM‐CSF macrophages and CD115 in M‐CSF/IL‐4 macrophages. GM‐CSF BMMs produced of significant levels of proinflammatory cytokines (IL‐1β, IL‐6, TNF‐α), which was Mincle‐dependent and further enhanced by LPS priming. M‐CSF BMMs produced little or no cytokines in response to TDB regardless of LPS priming. Western blot analysis confirmed that the absence of cytokine production was associated with a lack of activation of the Syk kinase pathway.

**Conclusion:**

This study illustrates that TDB has the potential to differentially regulate M1‐ and M2‐like macrophages in the tumor environment.

## Introduction

Innate immunity plays an important role in host protection against invading pathogens, and also in the progression of various diseases, such as autoimmunity, atherosclerosis, and cancer 1, 2. Accordingly, microbial products, such as trehalose glycolipids 3, have been widely studied for their ability to cause and treat disease 4, 5. Trehalose glycolipids were first identified in the cell wall of *Mycobacterium tuberculosis* 6, 7, and while the native isolated glycolipid, trehalose dimycolate (TDM), has been implicated in the pathogenesis of tuberculosis (TB) 8, both TDM and its C22‐acyl chain derivative, trehalose dibehenate (TDB), exhibit anti‐cancer activity and show promise as vaccine adjuvants 9, 10, 11, 12, 13, 14, 15.

TDM and TDB are recognized by the Macrophage Inducible C‐type Lectin (Mincle, Clec4e) 13, 16, and the activity of these glycolipids may be further enhanced through interaction with the macrophage C‐type lectin (MCL, Clec4d) 17, 18. Mincle is expressed on myeloid cells and recognizes damage‐associated molecular patterns (DAMPs) and pathogen‐associated molecular patterns (PAMPs) 16, 19. Upon binding of TDB to Mincle, the Syk (Spleen tyrosine kinase) and Card9–Bcl10–MALT1 inflammasome pathways are activated, which ultimately leads to the cellular production of cytokines and chemokines 20, 21, 22, 23.

The majority of studies on the activation of macrophages by TDM and TDB have focussed solely on inflammatory macrophages and dendritic cells (DCs) 13, 18, 20, 24, 25. Macrophages, however, exist as a continuum of phenotypes ranging from the classically activated “M1‐like” (inflammatory) to the alternatively activated “M2‐like” (anti‐inflammatory/wound healing) macrophage 26. Moreover, they display tremendous plasticity and readily alter their phenotype following exposure to changes in the local microenvironment 27. Understanding how these different phenotypes respond to agents such as TDB is key to understanding how best to exploit these cells in therapy.

In diseases such as cancer, different macrophage populations display distinct characteristics with often opposing immunological functions 28, 29. Macrophage progenitors exposed to a variety of immune regulatory cytokines, including IL‐4, can differentiate into alternatively activated (M2‐like) macrophages with tumor‐promoting properties 30, 31, 32. Thus, the ability to deplete tumor‐associated macrophages (TAMs) or convert them from an M2‐like phenotype to a more tumor‐suppressive phenotype represents a promising new approach for anti‐cancer therapies 32. As TDB and related trehalose glycolipids exhibit anti‐cancer activities and have been found to be effective adjuvants 9, 10, 11, 12, 13, 14, 15, we compared the effect of TDB on M1‐ and M2‐like macrophages.

## Materials and Methods

### Animals

C57BL/6 wild‐type mice and Mincle^−/−^ mice were bred and housed in a conventional animal facility at the Malaghan Institute of Medical Research, Wellington, New Zealand. All animals used for the experiments were aged between 8 and 12 weeks. All experimental procedures were approved by the Victoria University Animal Ethics Committee in accordance with their guidelines for the care of animals (protocol nr 22371).

### Generation and stimulation of bone marrow‐derived macrophages

Bone marrow cells were collected from the tibia and femur of C57BL/6 or Mincle^−/−^ mice and cultured (250,000 cells/mL) in complete RPMI media (RPMI‐1640 [Gibco, UK] with 10% heat inactivated fetal bovine serum [Gibco], 100 U/mL penicillin‐streptomycin [Gibco], and 2 mM Glutamax [Gibco]). Macrophage differentiation was induced by either 50 ng/mL GM‐CSF (PeproTech, Israel) or 10 ng/mL M‐CSF (PeproTech) with 10 ng/mL IL‐4 (PeproTech) added to the cRPMI 33, 34. Cells were incubated at 37°C (5% CO_2_) for 8 days (cells fed on days 3 and 6). Where indicated, BMMs were primed with 0.5 ng/mL LPS on day 7 for 24 h. On day 8, the media was removed and the cells were washed with DPBS to remove all non‐adherent and loosely adherent cells, and fresh complete RPMI was added to the cells followed by stimulation with 40 or 100 µg/mL TDB (2.5 mg/mL stock in DPBS with 2% DMSO), or 100 ng/mL LPS as positive control. TDB was synthesized according to previously published procedures 35 and was confirmed to be free of endotoxin at a sensitivity of ≤0.125 EU/mL by the Limulus amebocyte lysate (LAL) assay using an endotoxin kit (Pyrotell, MA, USA).

### Cytokine analysis

Levels of IL‐1β, IL‐6, and TNF‐α cytokines in the supernatants were determined by sandwich ELISA (BD Biosciences, CA, USA) according to the manufacturer's instructions.

### Quantitative RT‐PCR

GM‐CSF and M‐CSF/IL‐4 BMMs were differentiated over 8 days and stimulated with 100 µg/mL TDB for 48 h. Total RNA was extracted using Quick‐RNA™ MiniPrep kit (Zymo Research, CA, USA) followed by cDNA synthesis using iScript (BioRad, CA, USA) according to the manufacturer's instructions. Quantitative RT‐PCR of *18s*, *Cd74*, *Nos2*, *CD86*, *Chil3*, *Retnla*, and *Mrc1* (QuantiTect primer assay [Qiagen, Germany] and KAPA SYBR FAST qPCR Master Mix [x2] [Kapa Biosystems, MA, USA]) was performed using ABI 7500 platform. Cycle threshold (CT) was determined in the exponential phase of the amplification curve and CT of *Cd74*, *Nos2*, *CD86*, *Chil3*, *Retnla*, and *Mrc1* were normalized to the CT of *18s* ribosomal RNA (ΔCT). Amplification efficiency of QuantiTect primers are equivalent, so the ΔΔCT method was used to determine fold change (2^−ΔΔCT^). Results are expressed as log2 (fold change). All experiments were performed in triplicate.

### Western Blot

GM‐CSF and M‐CSF/IL‐4 BMMs were differentiated over 8 days and stimulated with 40 or 100 µg/mL TDB for 6 h. Whole cell lysate was collected with NP‐40 buffer containing protease (cOmplete Tablets, Roche, Switzerland) and phosphatase inhibitors (PhosSTOP, Roche). Western blots were performed by 10% SDS–PAGE and wet blotting. Antibodies used were anti‐P‐Syk(Y519/520), anti‐P‐Syk(Y317), anti‐Syk, anti‐β‐Actin, anti‐rabbit IgG‐HRP (Cell Signaling Technology, MA, USA) and anti‐P‐SHP2 (Y542), anti‐P‐SHP2(Y580) and anti‐SHP2 (Invitrogen, CA, USA). Blots were developed with SuperSignal West Femto Maximum Sensitivity Substrate (Thermo Scientific, IL, USA) and imaged with Amersham Imager 600.

### Flow cytometry analysis

Surface marker expression on macrophages was analyzed by flow cytometry (BD FACS Calibur and LSRFortessa Cell Analyser). Non‐specific staining was blocked using anti‐mouse CD16/32 antibody (2.4G2). The cells were then stained with fluorescently labelled antibodies for the surface markers I‐A/I‐E (MHC II) (Clone: M5/114.15.2, BioLegend, CA, USA), CD115 (Clone: AFS98, eBioscience, CA, USA), CD206 (Clone: CO68C2, Biolegend), F4/80 (Clone: BM8, BioLegend), CD11b (Clone: M1/70, BD Biosciences Pharmingen, CA, USA), CD86 (Clone: PO3, BD Biosciences Pharmingen), Streptavidin‐APC (BD Biosciences Pharmingen), and Mincle (Clone: 1B6, MBL International Corporation, Japan). Data analyses were performed using FlowJo (Tree Star, USA).

### Statistics

Statistical significance of differences was assessed using two tailed Student's *t*‐tests, 2‐way ANOVA with Bonferroni post‐hoc test, where appropriate, using Prism v7 software (GraphPad, CA, USA). A *P* value less than 0.05 was considered statistically significant.

## Results

### Verification of M1‐ and M2‐like macrophage populations

**Figure 1:**
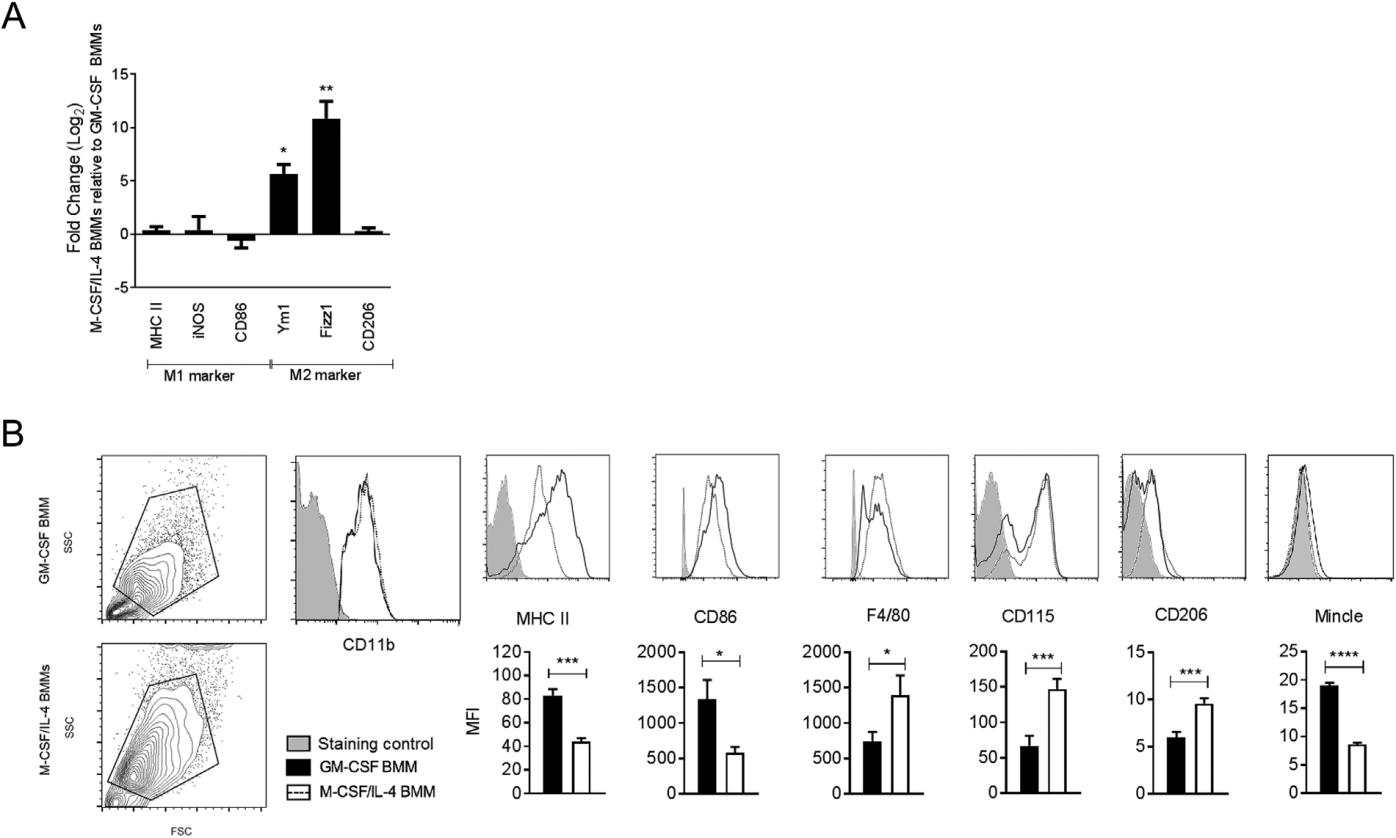
Differentiation of distinct M1‐ and M2‐like macrophages. (A) Gene expression profile of M‐CSF/IL‐4 BMMs as compared to GM‐CSF BMMs. M1‐like and M2‐like macrophages were generated using GM‐CSF and M‐CSF/IL‐4, respectively, over 8 days, and relative gene expression was measured by q/RT‐PCR. Mean ± SEM of triplicate samples from four independent experiments are shown. (B) Forward light scatter (FSC)—side light scatter (SSC) blots, macrophages were identified as CD11b positive cells. Cell surface expression of CD86, MHC II, F4/80, CD115, CD206, and Mincle on day 8 GM‐CSF and M‐CSF/IL‐4 differentiated BMMs as measured by flow cytometry. Unstained cells were used as a staining control for CD11b, MHC II, CD86, F4/80, CD115 and CD206, Mincle^−/−^ BMMs were used as a staining control for Mincle. Mean ± SEM of triplicate samples from at least three independent experiments are shown. *P ≤ 0.05; **P ≤ 0.01; ***P ≤ 0.005; ****P ≤ 0.001 (two‐tailed Student's *t*‐test).

First we confirmed the M1‐ and M2‐like phenotypes of the GM‐CSF and M‐CSF/IL‐4‐generated macrophage populations, respectively 26, 34, whereby the M‐CSF/IL‐4 BMMs are representative of TAMs as IL‐4 is often found in the tumor microenvironment 36, 37. The GM‐CSF and M‐CSF/IL‐4 BMMs expressed similar levels of M1‐like marker genes (MHC class II (*Ii*), iNOS (*NOS2),* CD86), while the M‐CSF/IL‐4 BMMs exhibited higher relative expression of the M2‐like markers Fizz1 (*Retnla*), Ym1 (*Chil3)*, and macrophage mannose receptor 1 “CD206” (*Mrc1*) (Fig. 1A). Analysis of cell surface marker expression on the two macrophage populations by flow cytometry showed that GM‐CSF BMMs expressed higher levels of MHC II, CD86 and Mincle, and lower levels of the M2 markers F4/80, CD115, and CD206 compared to M‐CSF/IL‐4 macrophages (Fig. 1B). Taken together, this data was consistent with the GM‐CSF and M‐CSF/IL‐4 BMMs exhibiting M1‐like pro‐inflammatory and M2‐like tissue‐resident anti‐inflammatory phenotypes, respectively 38, 39, 40.

### TDB activates GM‐CSF macrophages and depolarizes M‐CSF/IL‐4 macrophages

**Figure 2:**
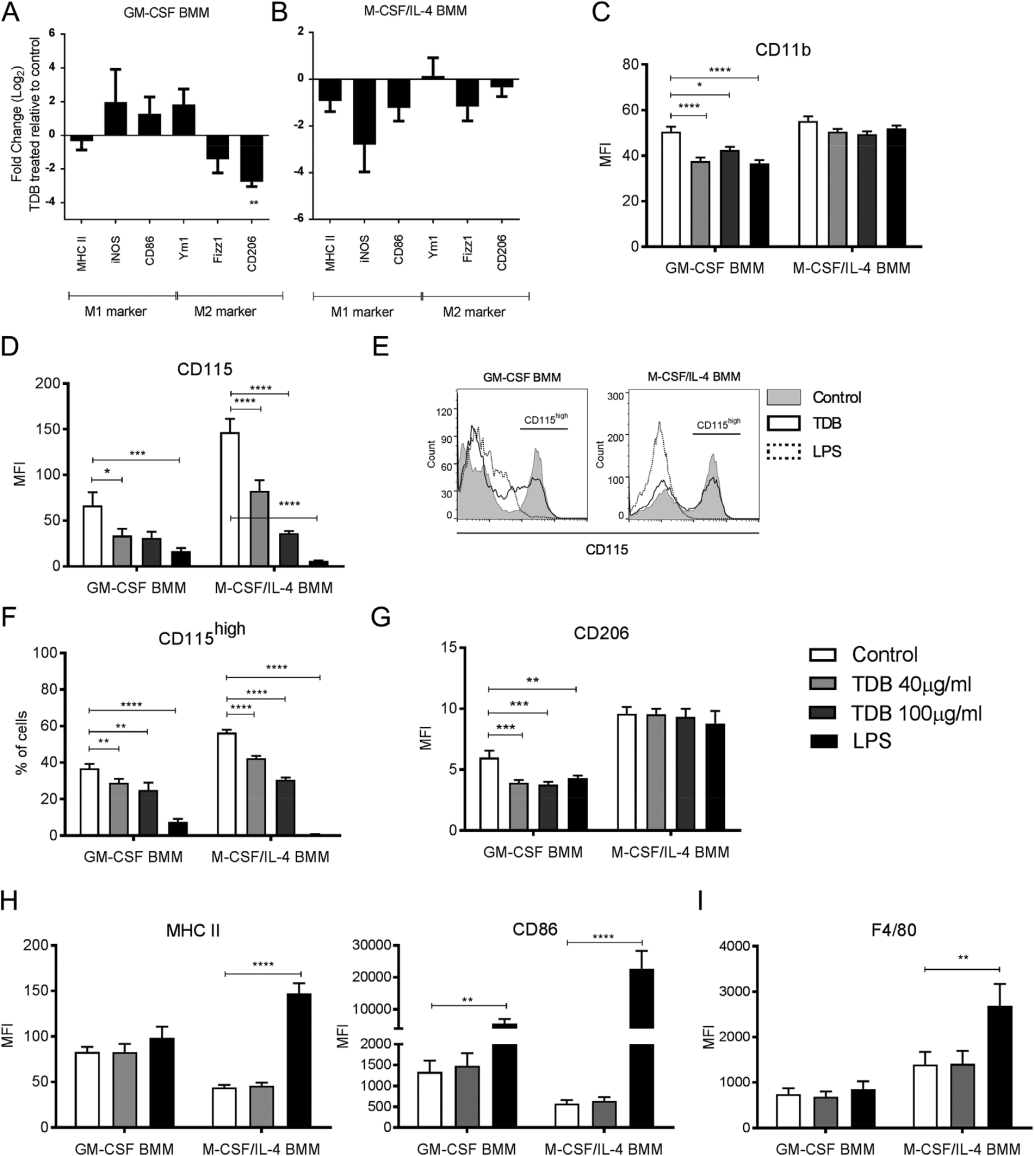
TDB changes the polarization of GM‐CSF and M‐CSF/IL‐4 BMM. Gene expression profile of TDB treated BMMs relative to untreated control. (A) GM‐CSF and (B) M‐CSF/IL‐4 BMMs were differentiated over 8 days, stimulated with 100 µg/mL TDB for 48 h, and relative gene expression was measured by q/RT‐PCR. Relative expression of cell surface markers (C) CD11b, (D) CD115, (G) CD206 and (H) MHC II, CD86, (I) F4/80 on GM‐CSF and M‐CSF/IL‐4 BMMs treated with 40 or 100 µg/mL TDB, or 100 ng/mL LPS as positive control, for 24 h. (E) The expression of CD115 by GM‐CSF and M‐CSF/IL‐4 BMMs shown as histogram and (F) the percentage of CD115high cells in TDB treated GM‐CSF and M‐CSF/IL‐4 BMMs. *P ≤ 0.05; **P ≤ 0.01; ***P ≤ 0.005; ****P ≤ 0.001 (two‐tailed Student's *t*‐test).

Macrophages often require a priming signal to boost their response to inflammatory stimuli 41. As TDB can deliver both priming and stimulatory signals 13, 20, 42, in this study TDB was added to the GM‐CSF and M‐CSF/IL‐4 BMMs without separate priming. Following TDB treatment, GM‐CSF BMMs exhibited a shift toward a more pro‐inflammatory phenotype, as indicated by the increased expression of iNOS and CD86 and the decreased expression of the M2 markers Fizz1 and CD206 (Fig. 2A). In contrast, M‐CSF/IL‐4 BMMs showed decreased expression of both M1‐ and M2‐like mRNA markers after TDB treatment (Fig. 2B).

Cell surface marker analysis showed that TDB treatment resulted in a significant decrease in the expression of CD11b for GM‐CSF BMMs (Fig. 2C) and lowered the expression of CD115 (Fig. 2D) for both macrophage phenotypes. Further analysis of CD115 expression on both GM‐CSF and M‐CSF/IL‐4 BMMs revealed a discrete CD115^high^ subpopulation (Fig. 2E) that decreased after exposure to TDB (Fig. 2F). Consistent with the mRNA results, TDB treatment also down regulated the relative expression of the M2‐like marker CD206 on GM‐CSF BMMs, but had no effect on the expression of CD206 on M‐CSF/IL‐4 BMMs (Fig. 2G). Although the activation of antigen presenting cells due to the uptake of mycobacteria is commonly associated with an increased expression of MHC class II and co‐stimulatory molecules such as CD86 43, TDB treatment did not alter the cell surface expression of MHC II or CD86 on either macrophage phenotype (Fig. 2H). TDB also had no effect on the expression of another commonly used murine macrophage marker F4/80 (Fig. 2I).

**Figure 3:**
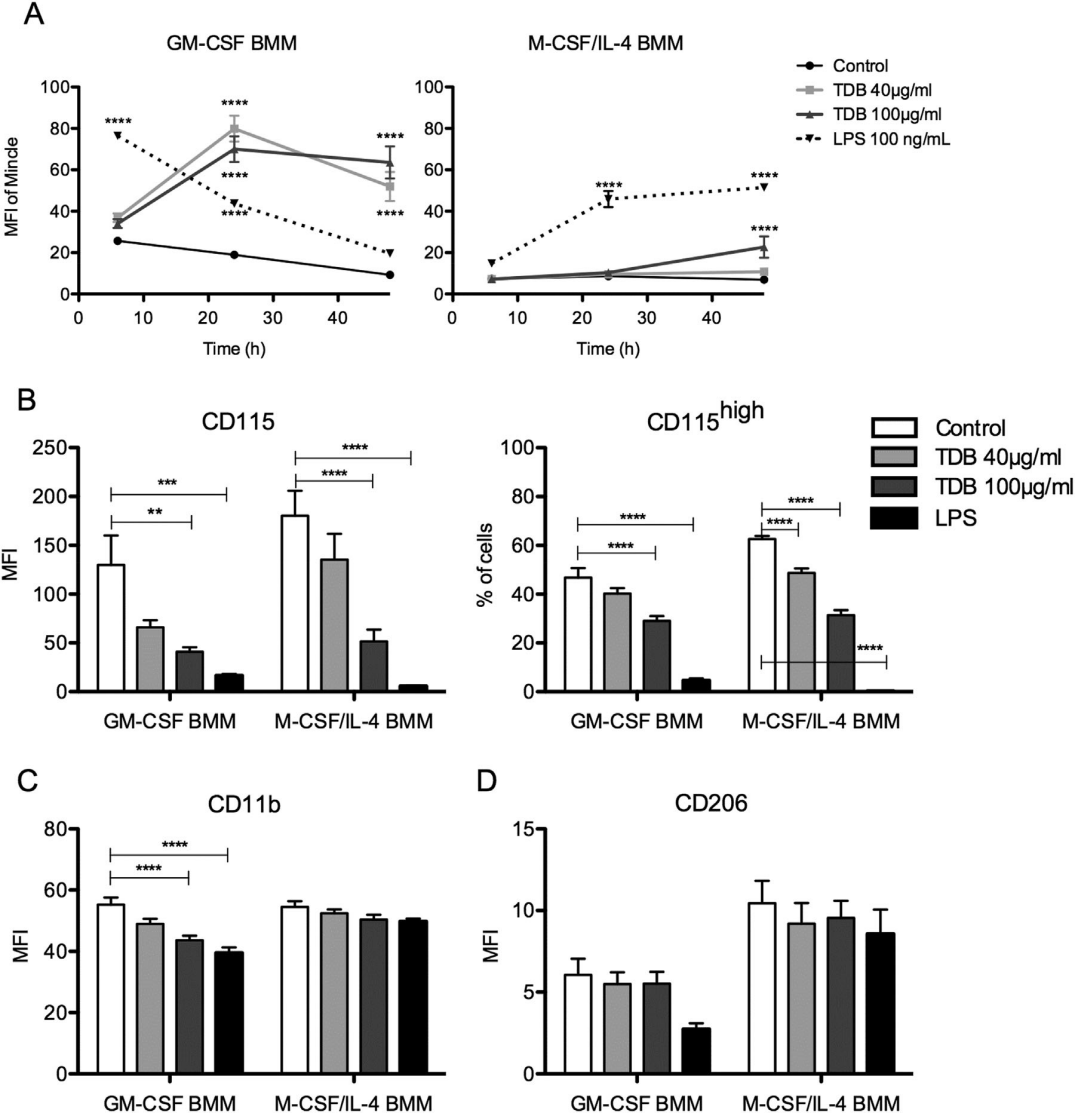
Effect of Mincle on surface marker expression by GM‐CSF and M‐CSF/IL‐4 BMMs. (A) Cell surface expression of Mincle in GM‐CSF and M‐CSF/IL‐4 differentiated BMMs from C57BL/6 bone marrow treated with 40 or 100 µg/mL TDB, or 100 ng/mL LPS as positive control, for 6, 24, and 48 h. Cell surface expression of (B) CD115 and percentage of CD115^high^ cells, (C) CD11b, (D) CD206 in GM‐CSF and M‐CSF/IL‐4 differentiated BMMs from Mincle^−/−^ bone marrow treated with 40 or 100 µg/mL TDB, or 100 ng/mL LPS as positive control, for 24 h. Mean ± SEM of triplicate samples from at least three independent experiments are shown. *P ≤ 0.05; **P ≤ 0.01; ***P ≤ 0.005; ****P ≤ 0.001 (two tailed Student's *t*‐test, 2‐way ANOVA with Bonferroni post hoc test).

Next, we looked at the effect of TDB treatment on changes to the cell surface expression of Mincle. Consistent with earlier findings 13, 18, TDB stimulated a rapid increase in Mincle expression on GM‐CSF BMMs (Fig. 3A). In contrast, the stimulation of M‐CSF/IL‐4 BMMs with a high concentration of TDB only led to a slight increase in Mincle expression over time. To determine whether the decreased expression of CD11b, CD115, and CD206 following TDB treatment required Mincle, BMMs were generated from Mincle^−/−^ bone marrow cells. The down regulation of CD115 expression for GM‐CSF and MCSF/IL‐4 BMMs (Fig. 3B) and CD11b for GM‐CSF BMMs (Fig. 3C) was still observed, indicating that Mincle was not necessary for inducing these effects. In the absence of Mincle, however, GM‐CSF BMMs did not down‐regulate CD206 following stimulation with TDB (Fig. 3D).

### TDB triggers pro‐inflammatory activation of GM‐CSF BMMs, but not M‐CSF/IL‐4 BMMs

**Figure 4:**
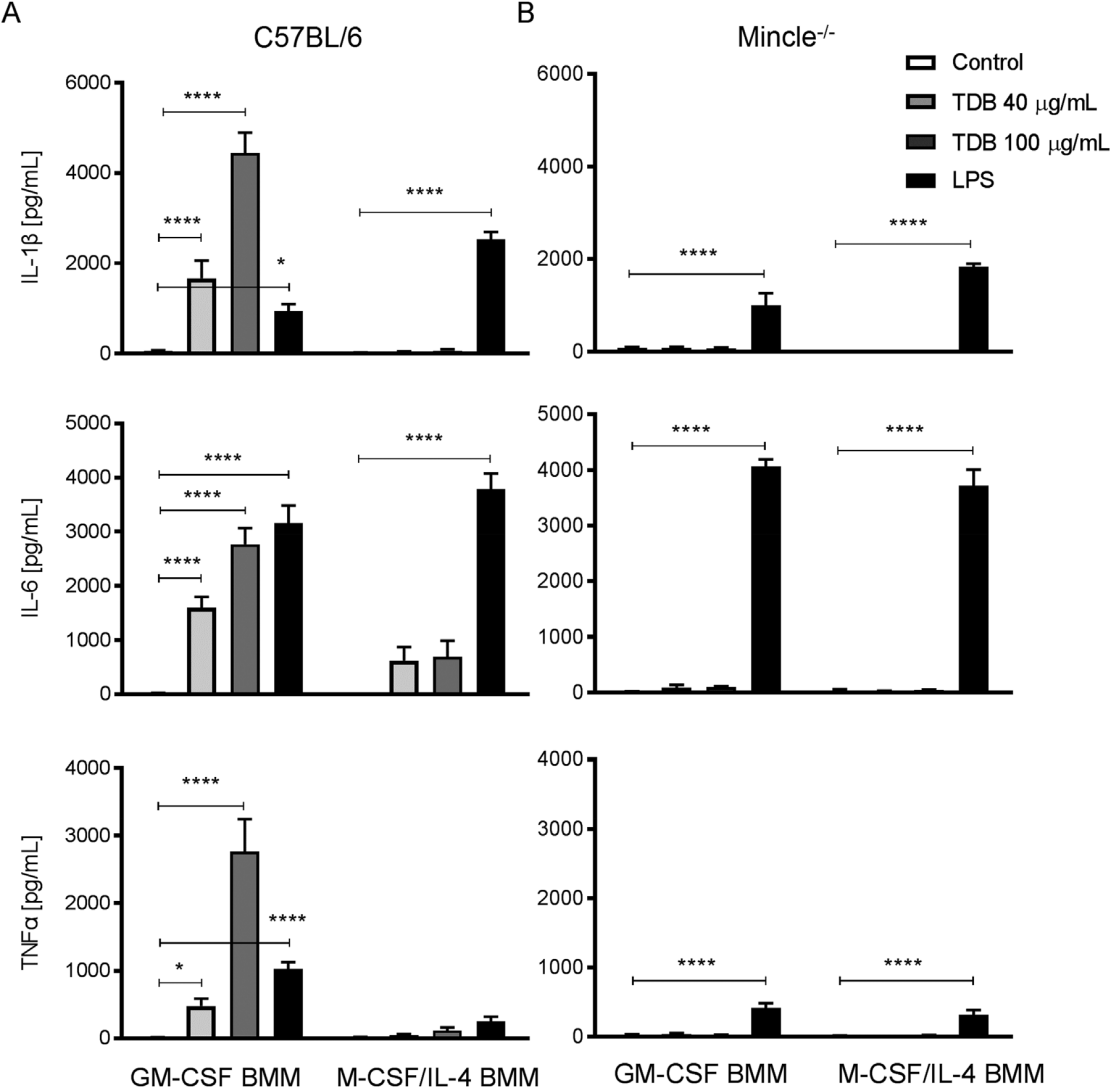
TDB triggers pro‐inflammatory activation of GM‐CSF BMMs, but not M‐CSF/IL‐4 BMMs. Cytokine production by TDB stimulated GM‐CSF and M‐CSF/IL‐4 BMMs from (A) C57BL/6 and (B) Mincle^−/−^ bone marrow. BMMs were treated with 40 or 100 µg/mL TDB, or 100 ng/mL LPS as positive control. Levels of IL‐1β, IL‐6, and TNFα were measured by the ELISA from supernatant at 48 h. Mean ± SEM of triplicate samples from at least three experiment performed are shown. **P ≤ 0.01; ***P ≤ 0.005; ****P ≤ 0.001 (2‐way ANOVA with Bonferroni post hoc test).

**Figure 5:**
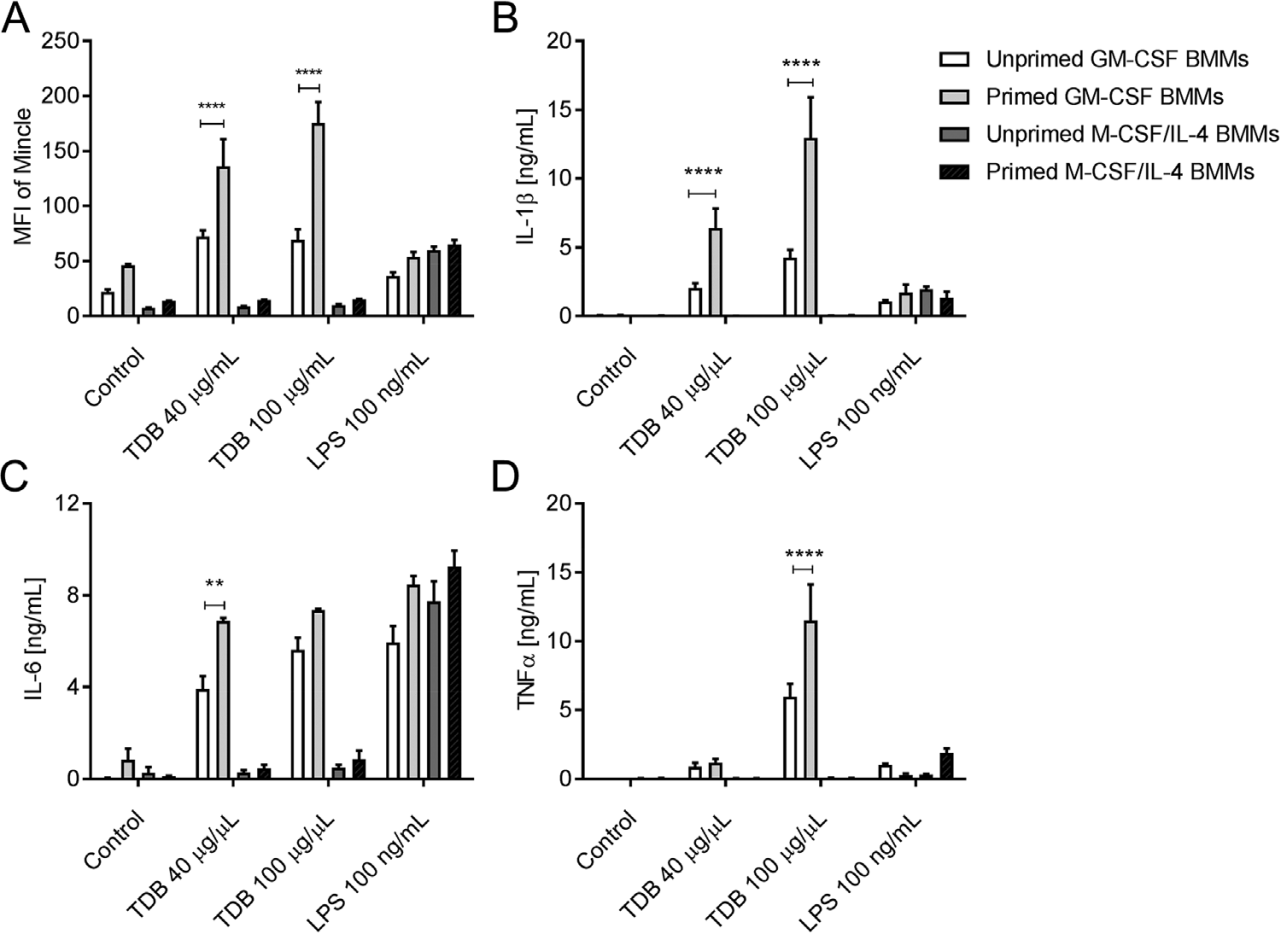
Priming does not increase M‐CSF/IL‐4 BMMs’ ability to respond to TDB. BMMs were differentiated with GM‐CSF and M‐CSF/IL‐4 from C57BL/6 bone marrow over 8 days, primed with 0.5 ng/mL LPS for 24 h and then stimulated with 40 or 100 µg/mL TDB, or 100 ng/mL LPS as positive control. Mincle expression (A) was measured at 24 h using flow cytometry, and production of IL‐1β (B), IL‐6 (C), and TNFα (D) measured by ELISA at 48 h. Mean ± SEM of triplicate samples from at least two experiment performed are shown. *P ≤ 0.05; ****P ≤ 0.001 (2‐way ANOVA with Bonferroni post hoc test).

We next explored the effect of TDB on the function of GM‐CSF and M‐CSF/IL‐4 BMMs. In accordance with previous studies using M1‐like macrophages 13, 20, 35, the exposure of GM‐CSF BMMs to TDB triggered the production of IL‐1β, IL‐6, and TNF‐α (Fig. 4A). As expected 13, this activity was abolished when using GM‐CSF BMMs from Mincle^−/−^ mice (Fig. 4B). Consistent with previous studies 23, 43, M‐CSF‐differentiated macrophages responded to TDB (Fig. SI1), however, TDB treatment induced little or no cytokine production by M‐CSF/IL‐4 polarized macrophages despite these cells still being able to respond to LPS (Fig. 4A). This observation is supported by earlier findings that show that IL‐4 down‐regulates the expression of Mincle in human and mouse macrophages and DCs 44. Priming of the macrophage populations with LPS enhanced TDB‐induced increases in both Mincle expression (Fig. 5A) and cytokine production by GM‐CSF macrophages, but did not affect the response of M‐CSF/IL‐4 macrophages (Fig. 5B–D). Priming of the macrophages with LPS also did not enhance the response of either macrophage phenotype to LPS (Fig. 5B–D).

**Figure 6:**
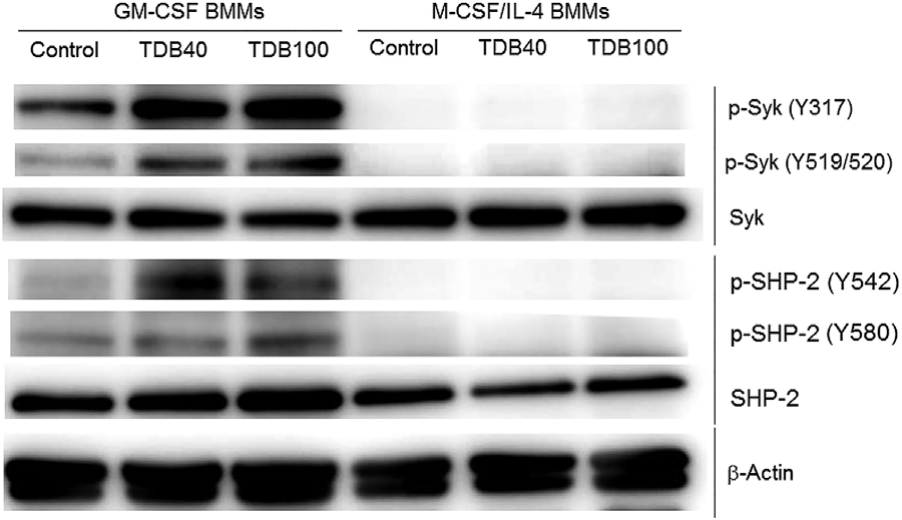
Syk kinase activity dependence of TDB‐induced inflammatory response by GM‐CSF and M‐CSF/IL‐4 BMMs. GM‐CSF and M‐CSF/IL‐4 BMMs differentiated from C57BL/6 bone marrow were stimulated with 40 or 100 µg/mL TDB for 6 h and whole cell lysate were analyzed for Syk and SHP‐2 phosphorylation by Western blot. β‐Actin was used as a loading control. Representatives of samples from at least two experiments performed are shown.

Phosphorylation of Syk 45 and the downstream formation of the Card9‐Bcl10‐Malt1 complex are essential for the activation of NFκB‐mediated gene expression via Mincle 20, 23, which in turn leads to pro‐inflammatory cytokine production and Mincle up‐regulation. To gain insight into the differences in cytokine production between the GM‐CSF and M‐CSF/IL‐4 macrophage populations, we analyzed signaling through the Syk‐Card9 pathway by western blot. Analysis of whole cell lysate showed comparable Syk expression in both GM‐CSF and M‐CSF/IL‐4 BMMs, however, only GM‐CSF BMMs showed Syk phosphorylation (Y317 and Y519/520) after TDB treatment (Fig. 6A and B). The tyrosine phosphatase SHP‐2 has been shown to mediate C‐type lectin receptor‐induced Syk activation 46, and western blot analysis showed that TDB treatment induced SHP‐2 phosphorylation (Y542 and Y580) in GM‐CSF BMMs alone (Fig. 6A and C). These data indicate that M‐CSF/IL‐4 BMM do not switch on the Syk‐Card9 pathway required to induce pro‐inflammatory responses via Mincle.

## Discussion

M1‐ and M2‐like macrophages display distinct characteristics and opposing immunological functions that can be targeted to treat disease 26. In this study, we demonstrate that TDB differentially modulates GM‐CSF (M1‐like) and M‐CSF/IL‐4 (M2‐like) macrophages. The effect of TDB on the two macrophage phenotypes is substantial, with TDB enhancing the M1‐like phenotype of GM‐CSF macrophages and down‐regulating M2‐like markers commonly associated with TAMs and cancer progression 31, 47, 48 for both GM‐CSF and M‐CSF/IL‐4 BMMs.

The ability of TDB to decrease the expression of CD115 may play a role in the reported anti‐cancer activity of the glycolipid. The accumulation of M2‐like TAMs is associated with a negative outcome in cancer 32, and accordingly, there has been much interest in targeting macrophages to block the development of this M2‐like phenotype 49, 50. Indeed, the inhibition of CD115 by monoclonal antibodies has been shown to inhibit TAMs and has proven to be a promising new anti‐cancer therapy 51, 52, 53, 54. Similarly, the observed decrease in CD11b following TDM treatment could also contribute to the anti‐cancer effects of TDB. CD11b has been correlated to a poor prognosis for certain cancers 55, and blocking CD11b has led to enhanced anti‐tumor therapies and outcomes 56, 57. The observed changes in the expression of CD115 and CD11b were independent of Mincle. We have previously reported that the cellular uptake of TDB is Mincle independent 58, and therefore it is possible that TDB is acting intracellularly to down regulate surface marker expression. Alternatively, other, yet unidentified, TDB receptors might be involved in these processes.

Consistent with previous work on M1‐like macrophages and DCs 13, 20, TDB‐treated GM‐CSF BMMs produced IL‐1β, IL‐6, and TNF‐α in a Mincle‐dependent manner. Similarly, M‐CSF BMMs also produced cytokines in response to TDB 23, 43. This proinflammatory response by GM‐CSF (M1‐like) macrophages, and M‐CSF BMMs, likely contributes to TDB's anti‐cancer properties as the immunological activity of both TDM and TDB has been linked with inflammation and lymphocyte sensitization 10. Notwithstanding, Mincle signaling has recently been associated with oncogenesis in pancreatic ductal adenocarcinoma (PDA) 59. However, PDA is characterized by inflammatory cells in the tumor microenvironment 60, while many other cancers contain high numbers of anti‐inflammatory macrophages 32. Accordingly, it is necessary to understand the tumor microenvironment and how different tumor macrophage populations are likely to respond to immunomodulating agents such as TDB in order to ensure that the appropriate local response is raised for the best therapeutic effect.

In contrast to GM‐CSF and M‐CSF BMMs, M‐CSF/IL‐4 BMMs did not produce inflammatory mediators in response to TDB despite showing the capacity to respond to LPS stimulation. Subsequent analysis of the cell surface expression levels of Mincle indicated that TDB induced rapid and significant increases in Mincle expression in GM‐CSF BMMs but not for M‐CSF/IL‐4 BMMs, which is consistent with the earlier findings by Hupfer et al. 44. Moreover, while LPS priming increased Mincle expression and cytokine production by GM‐CSF BMMs treated with TDB, LPS priming had no appreciable effect on Mincle expression or cytokine production by strongly M2‐polarized macrophages treated with TDB.

Our data on the activation of the Mincle‐Syk pathway also demonstrates that while both Syk and SHP‐2 proteins were phosphorylated in TDB‐treated GM‐CSF BMMs, this did not occur in M‐CSF/IL‐4 BMMs, with the likely explanation being the observed low expression levels of Mincle. However, based on work by Strasser et al. 61 showing that protein kinase C δ (PKCδ) links Syk activation by C‐type lectin receptors (including Mincle) to the Card9‐Bcl10‐Malt1 complex, a phosphoproteomic study could provide more insight into the Mincle‐driven signaling pathways for both macrophage phenotypes.

Taken together, our data demonstrates that TDB can modulate the immune response of M1‐like and M2‐like macrophages, with both macrophage subsets losing characteristics associated with an M2‐like phenotype. These functional changes are more pronounced for macrophages already skewed toward the pro‐inflammatory phenotype, with GM‐CSF BMMs becoming more pro‐inflammatory upon stimulation with TDB. The treatment of M‐CSF/IL4 BMMs with TDB, generates a more neutral macrophage phenotype, as evidenced by the decrease in the relative expression of CD115, deficit cytokine production, and an overall decrease in the expression of marker mRNA. Our data also suggests that TDB has the potential to alter macrophage phenotypes to one that disfavors tumor‐growth. Given that TDB is a well‐tolerated vaccine adjuvant, this work further supports the development of modified trehalose glycolipids with even more promising as anti‐cancer activity.

## Conflict of Interest

The authors declare that there are no conflicts of interest.

## Supporting information

Additional supporting information may be found in the online version of this article at the publisher's web‐site.

**Supporting Information SI1**. TDB induces the production of cytokines in M‐CSF differentiated bone marrow macrophages. BMMs from WT bone marrow were differentiated over 8 days using M‐CSF followed by stimulation with 40 μg/mL TDB, or 100 ng/mL LPS as positive control. Levels of IL‐1β, IL‐6, and TNFα were measured by the ELISA from supernatant at 48 h. Mean ± SEM of triplicate samples from one experiment are shown. *P ≤ 0.05; ****P ≤ 0.001 (1‐way ANOVA).
