# Supplementary material for: The Mincle ligand trehalose dibehenate differentially modulates M1‐like and M2‐like macrophage phenotype and function via Syk signaling
Source: Immun Inflamm Dis. 2017 Jul 19;5(4):503–14. doi: 10.1002/iid3.186 (PMC5691301; doi:10.1002/iid3.186)
Supplement: Supplementary file 1 — Supporting Information SI1. TDB induces the production of cytokines in M‐CSF differentiated bone marrow macrophages. BMMs from WT bone marrow were differentiated over 8 days using M‐CSF followed by stimulation with 40 μg/mL TDB, or 100 ng/mL LPS as positive control. Levels of IL‐1β, IL‐6, and TNFα were measured by the ELISA from supernatant at 48 h. Mean ± SEM of triplicate samples from one experiment are shown. *P ≤ 0.05; ****P ≤ 0.001 (1‐way ANOVA). [file IID3-5-503-s001.pdf]

SUPPORTING INFORMATION

The Mincle ligand trehalose dibehenate differentially modulates M1-like and M2-like macrophage phenotype and function via Syk signalling.

Kristel Kodar,[1,2] Jacquie L. Harper,[2] Melanie J. McConnell,[3] Mattie S. M. Timmer,[1,*] Bridget L. Stocker[1,2,*]

[1]School of Chemical and Physical Sciences, Victoria University of Wellington, PO Box 600, Wellington, New Zealand

[2]Malaghan Institute of Medical Research, PO Box 7060, Wellington, New Zealand

[3]School of Biological Sciences, Victoria University of Wellington, PO Box 600, Wellington, New Zealand

*Corresponding authors: Mattie S. M. Timmer and Bridget L. Stocker, School of Chemical and Physical Sciences, Victoria University of Wellington, PO Box 600, Wellington, New Zealand

Phone: +644 463 6529, +644 463 6481

Fax: +64 4 463 5241

E-mail: mattie.timmer@vuw.ac.nz, bridget.stocker@vuw.ac.nz

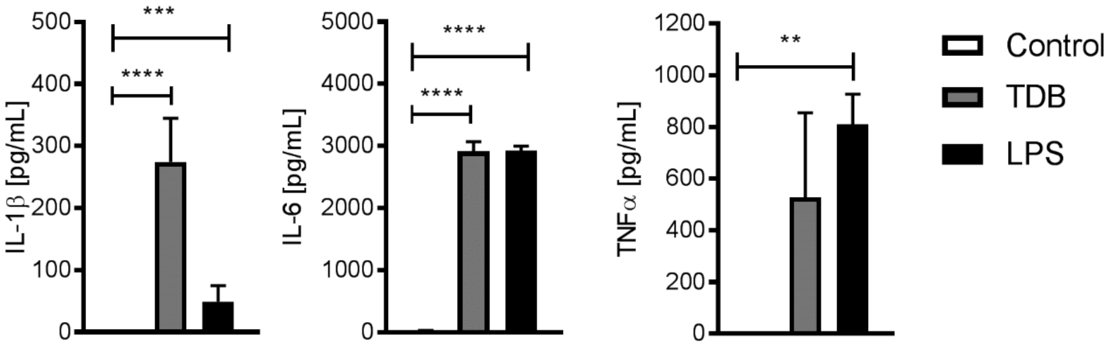

Figure SI1. TDB induces the production of cytokines in M-CSF differentiated bone marrow macrophages. BMMs from WT bone marrow were differentiated over 8 days using M-CSF followed by stimulation with 40 µg/mL TDB, or 100 ng/mL LPS as positive control. Levels of IL-1β, IL-6 and TNFα were measured by the ELISA from supernatant at 48 h. Mean ± SEM of triplicate samples from one experiment are shown. * $P \leq 0.05$; **** $P \leq 0.001$ (1-way ANOVA).
